# Study of Endothelio- and Osteoprotective Effects of Combination of Rosuvastatin with L-Norvaline in Experiment

**DOI:** 10.1155/2018/1585749

**Published:** 2018-11-05

**Authors:** M. S. Sobolev, A. V. Faitelson, O. S. Gudyrev, D. S. R. Rajkumar, G. M. Dubrovin, A. V. Anikanov, N. U. Koklina, E. S. Chernomortseva

**Affiliations:** ^1^Kursk Regional Children's Hospital No-2, No-43a, Khutorskaya Street, Kursk, Russia; ^2^Department of Traumatology and Orthopedics, Kursk State Medical University, No-3 Karl Marx Street, Kursk 305000, Russia; ^3^Department of Pharmacology, Belgorod State National Research University, 85 Pobedy Street, Belgorod, 308015, Russia

## Abstract

The experiment was carried out on 120 female white Wistar rats, to study the endothelio- and osteoprotective action of the combination of rosuvastatin with L-norvaline in the model of experimental osteoporosis. It was found that, after ovariectomy in rats, endothelial dysfunction of the vessels of the microcirculatory bed of bone tissue develops, leading to the appearance of osteoporosis, but the combination of the studied drugs prevents the decrease in the level of microcirculation in the bone tissue, thereby preventing the thinning of bone trabeculae and preventing the occurrence of microfractures in them.

## 1. Introduction

The main cause of development of osteoporosis is the imbalance of the key processes: process of bone resorption and remodeling [1]. It is known that the blood supply plays a significant role in the regeneration of bone tissue. Deterioration of blood supply leads to a decrease in the number of osteoblasts and inhibits their activity, while osteoclasts activate their activity. Dysfunction of the endothelium of microcirculatory vessels, most often, is the basis in the pathogenesis of reducing microcirculatory blood flow in the bones [2], which, in turn, leads to a disturbance of osteogenesis, thereby causing osteoporotic changes in bone tissue [3].

In a number of experimental studies, the positive effects of drugs possessing endotheliotropic properties on repair and regeneration of bone tissue have been proved. These include enalapril, losartan, and resveratrol [4–6]. It can be assumed that, in addition to these drugs, a combination of drugs consisting of rosuvastatin and L-norvaline possesses the same endothelioprotective properties. Under the influence of this combined treatment, the flow of blood in the microcirculation is normalized, leading to a slowdown in the development of osteoporotic changes in the spongy bone tissue. At present, there is no evidence in modern scientific publications that this combination of drugs has been used in the pharmacotherapy of osteoporosis; this confirms the relevance of the research topic.

## 2. Objective

The objective of this paper is the analysis of the effectiveness of pharmacological correction of experimental osteoporosis with a combination of rosuvastatin with L-norvaline compared with drugs strontium ranelate (Bivalos) and Calcium D3 Nycomed.

## 3. Materials and Methods

All experimental manipulations were performed under general anesthesia by intraperitoneal injection of a solution of chloral hydrate at a dose of 300 mg/kg. Modeling of hypoestrogenic osteoporosis was done by performing bilateral ovariectomy surgery to female Wistar rats of weight 200–250 g [2, 6]. After 56 days, in all the animals in the spongy bone tissue of the trochanteric region of the femur bone were formed hypoestrogen-induced osteoporotic changes (according to the results of histomorphometry).

The animals were divided into 5 groups. In the control group (n=20), the rats underwent an operation: false ovariectomy (without removal of the ovaries). In group II (n=20), the animals underwent surgery, bilateral ovariectomy (osteoporosis model). As a placebo this group received for eight weeks daily intragastrical paste of 1% starch.

In the rats of group III (n=20), after modeling of hypoestrogenic osteoporosis, after eight weeks, a combined treatment with rosuvastatin 0.86 mg/kg and L-norvaline at a dose of 10 mg/kg for four weeks was given. The animals in groups IV and V (20 rats in each group) received, respectively, Calcium D3 Nycomed at a dose of 85.7 mg/kg and strontium ranelate (Bivalos) at a dose of 171 mg/kg from the ninth to the twelfth week. The studied drugs were injected intragastrically daily as a suspension in 1% starch paste.

After 12 weeks in the experimental animals, the index of intraosseous microcirculation was measured in the trochanteric region of the femur and the coefficient of endothelial dysfunction (CED) was calculated on the basis of laser Doppler flowmetry (LDF) data. Calculation and registration of parameters of microcirculation and CED were performed using a laser Doppler flowmetry Biopac system MP-100 and a needle-shaped sensor TSD-144; the results obtained were processed by the AcqKnowledge version 3.8.1-4.2.0 program. The values of microcirculation were expressed in perfusion units (PU) and CED in conventional units (standard units).

To assess the formation of osteoporotic disorders and the effectiveness of therapy of the studied drugs, histomorphometry of the proximal metaphysis of the femur was performed. When calculating the width of the bone trabecula, ImageJ version 1.39 was used. The values obtained were expressed in micrometers (*μ*m).

## 4. Results 

When measuring the parameters of intraosseous perfusion in the trochanteric region of the femur in rats, the following was found:

- In the control group (false ovariectomy): 99.91±3.41 PU
- In group II (model of osteoporosis without treatment): 58.75±3.76 PU
- In group III (combination therapy with rosuvastatin and L-norvaline): 88.02±3.03 PU
- In group IV (therapy with Calcium D3 Nycomed): 56.89±2.02 PU
- In group V (therapy with strontium ranelate): 67.48±2.98 PU

Thus, it was found that the combination of rosuvastatin and L-norvaline in the studied dosages increased the rates of regional intraosseous microcirculation of the proximal femur. Data of LDF-graphs in a group of rats that received a combination of the studied drugs were statistically significantly different from those of animals with osteoporosis, as well as rats treated with the drugs of comparison: Bivalos and Calcium D3 Nycomed.

To clarify the role of endothelial dysfunction (ED) in the formation of intraosseous microcirculation disorders, functional vascular tests were performed: one-time intravenous injection of solutions of acetylcholine and nitroprusside (at doses of 40 *μ*g/kg and 30 *μ*g/kg, respectively). With the introduction of these drugs, there was a decrease in the microcirculatory blood flow, followed by normalization of the microcirculation indices. The reason for this phenomenon is that acetylcholine and nitroprusside cause systemic vasodilation; that is, there is centralization of blood flow and, consequently, a decrease in the velocity of blood flow volume in bone tissue. The calculation of this coefficient was carried out on the basis of the LDF-graph that is defined as the ratio of the area of the triangle above the microcirculation recovery curve in response to the introduction of nitroprusside to the area of the triangle above the microcirculation recovery curve in response to the introduction of acetylcholine.

In the control group the value of CED was 1.28±0.18; in the group of rats with experimental osteoporosis CED was 2.57±0.23. These results indicate the formation of changes leading to dysfunction of the endothelium of the blood channel in the bone tissue after ovariectomy. When administering combined treatment with these drugs, a statistically significant decrease in the coefficient of 1.68±0.25 was found. Calcium D3 Nycomed and the strontium ranelate statistically significant changes in this indicator were not found (2.64±0.17 and 2.44±0.19, respectively). The obtained data indicate that the combination of the drugs under study, in comparison with Calcium D3 Nycomed and strontium ranelate, has endothelioprotective activity. This property of this combination has a positive effect on intraosseous microcirculation, which results in the stabilization of bone remodeling processes, which prevents the progression of the formation of osteoporotic disorders in the spongy bone tissue.

**Figure 1:**
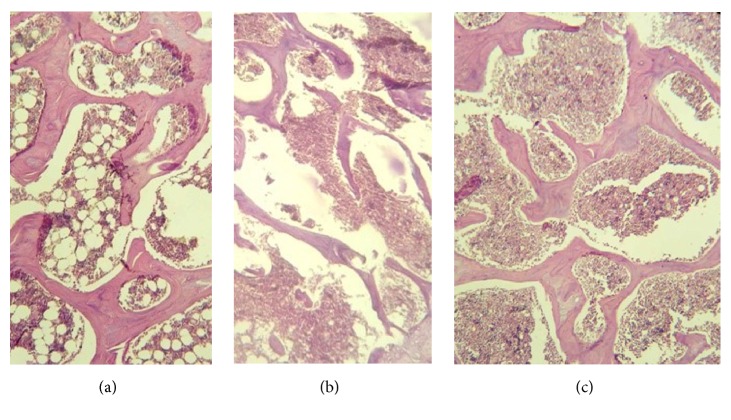
The histological picture of proximal metaphysis of the femoral bone (strained with hematoxylin and eosin, zoom, x100). (a) Control group; (b) group with a model of osteoporosis, without treatment; (c) group treated with L-norvaline.

This position is confirmed by the results of histological studies. Microscopic morphological sections of the trochanteric zones of the rat's femur that received combined treatment of rosuvastatin and L-norvaline are determined by the normal microarchitecture of the bone tissue in comparison with the animals with osteoporosis (Figure 1).

An objective evaluation criterion for the formation of osteoporotic shifts and the effectiveness of the treatment after bilateral ovariectomy is histomorphometry. The mean width of the bone trabeculae in the localization was calculated. Thus, a significant decrease in the average width of bone trabeculae in animals with a model of osteoporosis (64.61±0.54 *μ*m) was found to be 33% higher than in the rats of the control group (96.64±1.01 *μ*m).

In a morphometric study, it was found that the combination of rosuvastatin with L-norvaline in the previously indicated dosages prevented a decrease in the average width of the bone trabeculae to a level of animals with experimental osteoporosis by 30% (84.02±0.89 *μ*m). However, the average width of the trabeculae did not reach the values of the control rats but statistically significantly exceeded the parameters obtained in animals treated with comparative drugs: strontium ranelate (80.19±0.95 *μ*m) and Calcium D3 Nycomed (58.23±0.97 *μ*m).

Thus, the studied combination of drugs, three months after the modeling of osteoporosis in rats, has a protective effect, which is slowing down the thinning of the trabeculae in the trochanteric region of the femur.

## 5. Discussion

The endothelial layer of the intraosseous vessels is an integral part of the bone and plays a central regulatory role [7], with significant metabolic activity, while performing various functional activities, which include regulation of leukocyte adhesion, regulation of vascular growth, atrombogenicity and thrombogenicity of the vessel wall, and immune functions [8]. Endothelial cells themselves produce a variety of biologically active substances that are involved in the regulation of vascular tone [9]. A significant number of mediators that are produced by the endothelial layer are vasoconstrictors, angiotensin II and endothelin I, and vasodilators, nitric oxide (NO), endothelial hyperpolarizing factor, and prostacyclin [10]. Endothelial dysfunction is an imbalance between vasodilating and vasoconstrictor mediators, which is characterized by a decrease in the production of vasodilators with activation of the synthesis of vasoconstrictors. The primary vasodilating mediator is nitric oxide, which as a result of biosynthesis is extracted from the amino acid of L-arginine with the participation of the endothelial nitric oxide synthase (NO synthase) enzyme [11]. Decrease in expression or transcription of NO synthase and a decrease of L-arginine reserves availability, as well as the acceleration of nitric oxide metabolism, lead to a disruption of its synthesis and, as a result, endothelial dysfunction occurs. The metabolism of L-arginine in the cells proceeds in two ways: the first one: under the action of the digestion, it is hydrolyzed to ornithine and urea; the second: transformation: L-arginine is hydrolyzed to nitric oxide and citrulline, and it is catalyzed by NO synthase. The enzymes arginase and NO synthase compete with each other for a common substrate: L-arginine [10]. In a number of studies, there was an increase in the activity of arginase with the development of endothelial dysfunction. Also, arginase inhibits the activity of nitric oxide synthase, inhibiting the production of nitric oxide. The decrease in the effect of arginase leads to an increase in the production of nitric oxide, favorably affecting the normalization of vascular function [11].

From the foregoing, it can be concluded that the use of arginase inhibitors is necessary to increase the production of nitric oxide, which helps prevent the formation of endothelial dysfunction. Arginase inhibitors (such as L-norvaline) block the enzyme arginase, while most likely preventing metamorphosis of L-arginine in urea and ornithine. Due to this, a significant amount of L-arginine is cleaved under the influence of NO synthase by the formation of NO. This positively affects the regional microcirculation of the intraosseous structures, maintaining within a reasonable range the homeostasis in the bone tissue.

Another group of drugs, which is very popular in the complex treatment of vascular pathology, are statins that can improve endothelial function. Their pharmacological actions are based on the fact that lipids normally cannot penetrate through the intima of the vessels. When the barrier function of the endothelium is violated, systemic and local inflammatory mediators are produced under the influence of risk factors (smoking, hypercholesterolemia, hyperinsulinemia, arterial hypertension, menopause, etc.), leading to a decrease in the stability of endothelial NO synthase that is leading to the development of endothelial dysfunction [12]. In experimental models of hypercholesterolemia in pigs, it was found that statins have a positive effect on the function of endotheliocytes and improve the myocardial perfusion. The improvement in endothelial function does not depend on the concomitant decrease in cholesterol level [13]. Preparations of this group can increase the activity of endothelial NO synthase in the culture of human endothelial cells by stabilizing its mRNA. Subsequently, it was found that statins stimulate the expression of endothelial NO synthase mainly due to the effect on posttranscriptional mechanisms.

These substances also have other mechanisms of action on the activity of endothelial NO synthase: they activate protein kinase in endothelial cells, which in turn leads to phosphorylation of endothelial NO synthase, which increases its activity and stimulates NO synthesis. Also, there is the ability of these drugs to increase the production of NO, while inhibiting the expression of caveolin-1, which can reduce the activity of endothelial NO synthase by binding to this enzyme. This fact is confirmed by the results of experimental studies in which it was clearly demonstrated that statins improve cerebral blood flow and have a cardioprotective effect with an indirect increase in the secretion of NO in the endothelium, independent of their hypolipidemic action.

Statins have the ability to increase the activity of endothelial NO synthase. Stimulation of NO production in the endothelium is a characteristic of all statins and does not depend on their effect on cholesterol synthesis [13, 14].

Moreover, the use of modern statins, including rosuvastatin, positively affects the elastic properties of the vascular wall and reduces endothelial dysfunction, increasing the functional activity of endotheliocytes. Stimulation of NO production in the endothelium is a characteristic of all statins and does not depend on their effect on the synthesis of cholesterol. These properties have a positive effect on the state of intraosseous microcirculation, therefore indirectly improving the trophism of bone tissue, including positively affecting osteoregeneration.

In view of the foregoing, it can be assumed that L-norvaline and rosuvastatin, despite having different pharmacokinetic mechanisms of action, potentiate the actions of each other when they are in combined therapy, which positively affects the process of bone remodeling.

## 6. Conclusion

1. The combination of rosuvastatin with L-norvaline in the indicated dosages on the chosen model of pathology has a significant endothelioprotective effect. It is manifested by the decrease in the coefficient of endothelial dysfunction, which predisposes to the development of microcirculatory disorders
2. The combination of rosuvastatin with L-norvaline effectively prevents the decrease in blood flow in the bone tissue of the trochanteric region of the femur, keeping it at a level close to animals without osteoporosis
3. The combination of rosuvastatin with L-norvaline has more pronounced osteoprotective effect than strontium ranelate and Calcium D3 Nycomed
